# Carbon migration and metagenomic characteristics during anaerobic digestion of rice straw

**DOI:** 10.1186/s13068-020-01770-4

**Published:** 2020-07-20

**Authors:** Dadi Chen, Xiaoyu Zuo, Juan Li, Xitong Wang, Jie Liu

**Affiliations:** 1 Department of Environmental Science and Engineering, College of Chemical Engineering, Beijing University of Chemical Technology, 15 BeiSanhuan East Road, ChaoYang District, Beijing, 100029 People’s Republic of China; 2 Beijing Municipal Research Institute of Environmental Protection, Beijing, 100037 People’s Republic of China; 3 Beijing Municipal Ecological and Environmental Monitoring Center, 14 Chegongzhuang West Road, Haidian District, Beijing, 100048 People’s Republic of China

**Keywords:** Anaerobic digestion, Straw, Process parameter, Metagenome, Metabolic pathway, Methane metabolism

## Abstract

**Background:**

Considerable interest has been expressed in the development of anaerobic digestion (AD) of straw to solve the environmental problems caused by the dumping and burning of straw and to generate clean energy. However, the poor biodegradability of straw and the low efficiency of energy generation achieved during its AD are problematic. Studying the parameter changes involved in the process of AD is helpful for clarifying its micro-mechanisms and providing a theoretical basis for improving its efficiency. Currently, most research into process parameters has focused on gas production, methane content, pH, and volatile fatty acid (VFA) content; limited research has focused on carbon migration and functional gene changes during the AD of straw.

**Results:**

Carbon migration and changes in metagenomic characteristics during the AD of rice straw (RS) were investigated. Accumulated biogas production was 388.43 mL/g VS. Carbon in RS was consumed, and the amount of carbon decreased from 76.28 to 36.83 g (conversion rate 51.72%). The degree of hydrolysis rapidly increased during the first 5 days, and a large amount of carbon accumulated in the liquid phase before migrating into the gas phase. By the end of AD, the amount of carbon in the liquid and gas phases was 2.67 and 36.78 g, respectively. According to our metagenomic analysis, at the module level, the abundance of M00357, M00567, M00356, and M00563 (the modules related to the generation of methane) during AD were 51.23–65.43%, 13.96–26.88%, 16.44–22.98%, and 0.83–2.40%, respectively. Methyl-CoM, 5-methyl-5,6,7,8-tetrahydromethanopterin, and Acetyl-CoA were important intermediates.

**Conclusions:**

Carbon was enriched in the liquid phase for the first 5 days and then gradually consumed, and most of the carbon was transferred to the gas phase by the end of AD. In this study, AD proceeded mainly via aceticlastic methanogenesis, which was indicated to be a dominant pathway in methane metabolism. Batch AD could be divided into three stages, including initiation (days 1–5), adaptation (days 6–20), and stabilization (days 21–50), according to biogas production performance, carbon migration, and metagenomic characteristics during AD.
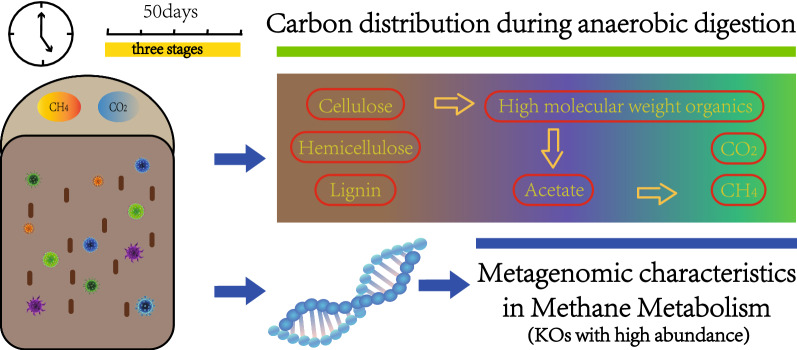

## Background

In recent years, anaerobic digestion (AD) technology has been recognized as an environmentally friendly method to control greenhouse gas emissions and produce clean energy in the form of biogas [1]. AD technology can address the environmental issues caused by the dumping and burning of straw and exhibits immense potential for the production of biogas with high methane content [2]. The efficient conversion of straw to methane is limited by the recalcitrant lignocellulosic structure of straw, resulting in the low susceptibility of its lignocelluloses to hydrolysis [3]. However, these problems of poor biodegradability and low efficiency of AD when using straw have been widely researched [3–5], whereas research into the fundamental process changes involved in AD is limited.

To elucidate the mechanism of AD, many researchers have focused on process parameters as AD proceeds. For instance, Boe et al. monitored the process parameters of biogas production, methane content, pH value, and volatile fatty acid (VFA) content in the AD of cow dung and analyzed the acetic acid and propionic acid content; the results indicated that a high organic loading rate could inhibit the AD process [6]. Lv et al. monitored biogas production, methane content, pH value, VFAs, and other process parameters in a continuous stirred tank reactor (CSTR) used for the AD of corn silage and found that ammonia inhibition occurred in the reactor [7]. Li et al. investigated biogas production, biogas components, pH values, VFAs, ammonia nitrogen concentration, and other process parameters in a CSTR system used for the AD of vegetable waste and found that the reaction was inhibited due to its high organic loading rate [8]. However, most of these studies of process parameters focused on CSTRs, and the main parameters investigated were biogas production, methane content, pH value, VFAs, and ammonia nitrogen concentration. Carbon is an important component of straw. In AD, solid carbon in straw is first hydrolyzed and transformed into a liquid state before being transformed into CH_4_ and CO_2_ by microorganisms. The proportion of carbon converted differs among the three phases of the AD process. The conversion law of carbon elements in the AD process can provide a basis for the further clarification of the law of AD of straw and should be investigated. However, relatively few studies have investigated carbon migration and changes in parameters during AD of straw.

As promoters of AD, microorganisms play a key role in this process. Many researchers have conducted considerable research into the microbial community involved in the AD system. Chen et al. (2017) reported that *Euryarchaeota* and *Firmicutes* formed the dominant microbial communities at pH levels of 7–8 at high temperatures, with abundances of 58–60% and 19–23%, respectively, and the abundance of *Firmicutes* reached 60% when the pH increased to 9 [9]. Cai et al. discovered that the addition of Fe, Co, Ni, Mo, Se, or Mn during the AD of straw affected the relative abundance of bacteria and archaea in the system [4]. Zhou et al. [10] demonstrated that *Firmicutes* were the dominant bacteria in a two-phase system, whereas *Proteobacteria* were the dominant bacteria in a one-phase system [11]. However, studies of microbial communities during AD have mainly focused on comparisons among different AD conditions, and no reports have investigated the microscopic mechanisms during batch AD processes. According to the literature, the succession of microbial communities during the AD process is very complex; therefore, metagenomics may be used as an alternative approach. Wang et al. conducted a metagenomic analysis of the AD of weed silage, and the results showed that the composition, structure, and abundance of dominant bacterial communities in an AD system changed under different environmental conditions [11]. Fontana et al. performed a metagenomic study of one- and two-phase thermophilic AD of cheese wastewater; the results showed that the microbial community structures were significantly different between the two types of AD reactors and that increased microbial diversity was observed in the two-phase AD reactors [12]. Zhang et al. found that a high concentration of ammonia nitrogen was beneficial for the accumulation of VFAs but can inhibit Acetyl-CoA and Methyl-CoM gene expression, thereby significantly inhibiting the methane metabolism pathway [13]. Hu et al. analyzed the biodegradation pathway in an optimized activated carbon-containing AD reactor using Kyoto Encyclopedia of Genes and Genomes (KEGG) analysis and found that the abundance of the pathways involved in the conversion of propionic acid into acetic acid was increased in the activated carbon-enhanced reactor and that the pathways involved in lipid and methane metabolism were also enhanced [14]. However, most metagenomic analyses exclusively focused on microbial communities during digestion; in addition, the succession of metabolic pathways during the AD process has not been revealed to date.

The laws of carbon migration and knowledge of the functional gene changes during the AD of straw will provide a new perspective for the study of the micro-mechanisms that occur in this process. Therefore, the present study focused on carbon migration and metagenomic characteristics during the AD of rice straw (RS) to elucidate this process from the perspectives of the conversion of materials and functional characteristics. The aim of the present study was to present a thorough understanding of the AD of straw and provide a theoretical reference for further research into improving the performance of AD of straw.

## Results and discussion

### Biogas production performance

**Fig. 1:**
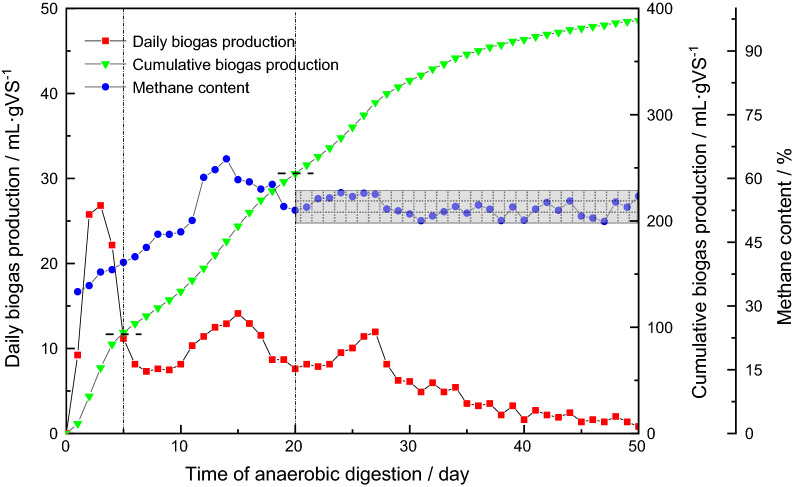
Daily and cumulative biogas production and methane content of rice straw during anaerobic digestion. ^a^The highlighted section indicates that the methane content had reached a stable state. ^b^The three stages of the anaerobic digestion process are indicated by vertical dotted lines

The biogas-producing performance of the AD of RS is shown in Fig. 1. Three peaks in gas production were noted during the AD process. The first peak in daily biogas production occurred on day 3, the second peak occurred on day 15, and the third peak occurred on day 27. During the first peak, the volume of gas produced was 26.81 mL/g VS; however, the methane content was low at 33.37%. During the second peak, the volume of gas produced was 14.12 mL/g VS, and the methane content was 59.73%. During the third peak, the volume of gas produced was less than that produced in the previous two peaks at 11.95 mL/g VS with the methane content of 56.30%. These results are consistent with those reported by Hu et al., who showed that three peaks appeared as the digestion process proceeded with the production of biogas decreasing in sequence [15]. The methane content gradually increased from 33.37% on day 1 to a maximum value of 64.59% by day 14; thereafter, the levels began to decline, reaching a stable state after day 20 and remaining within the range of 49.89% to 56.51%, which is highlighted in Fig. 1. The cumulative volume of gas produced by the end of the AD was 388.43 mL/g VS, which is similar to the results reported by Dai et al. [16].

The pH level, ammonia nitrogen content, and total alkalinity are important indexes that can be used to evaluate the stability of anaerobic digestive systems and microbial metabolism. The pH, ammonia nitrogen content, and total alkalinity of the liquid discharged following AD were measured. The pH of the liquid was 7.33, and the alkalinity was 4000 mg/L. Both of these values were within the normal range (pH > 6.8, alkalinity > 2000 mg/L). However, the ammonia nitrogen content was 358 mg/L, which did not exceed the tolerance range of anaerobic microorganisms (2 g/L). The methane production process was not inhibited [17], and the system reached a stable state following AD.

The biogas-producing performance of the AD of RS determined in the present study was similar to other results reported in the literature [18, 19]. These results indicate that the AD process was successful, and subsequent analysis based on this study is justified. If the analysis was performed by considering 5-day units, the AD process can be divided into three stages, which are indicated by the vertical dotted line in Fig. 1. The daily biogas production during the first unit of digestion time exhibited large fluctuations. The methane content gradually increased, and the first rapid increase in cumulative biogas production occurred. This increase may be attributed to the fact that bacteria are more active than archaea at the beginning of AD, leading to the accumulation of a large amount of acidic substances and inhibiting the progression of methane production. During the next three units of digestion time, the range in the fluctuations of daily biogas production decreased. The methane content increased to the maximum observed value in the present study and subsequently decreased to 56.1%, and the cumulative biogas production rapidly increased for the second time. This process potentially occurred due to the gradual acclimatization of the microorganisms to the system and stabilization of the synergy among the microorganisms. During the remainder of the unit digestion time, the daily biogas production decreased following the transitory increasing period and finally approached zero. The methane content tended to be stable, and the cumulative biogas production increased gradually after the third period of rapid increase. This finding could be attributed to the relatively stabilized microbial composition; however, the amounts of components that could be used for biological purposes in the RS raw material gradually decreased, and finally AD ceased.

### Distribution of carbon among the three phases

**Fig. 2:**
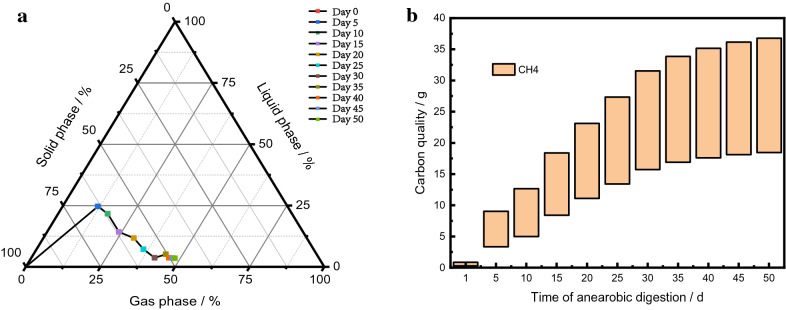
The distribution of carbon in the solid, liquid, and gas phases (**a**) and the distribution of carbon in the gas phase (**b**) during the anaerobic digestion of rice straw

Total organic carbon (TOC) reflects the amount of carbon in the liquid phase during AD because almost all the carbon in the liquid phase exists in the form of organic carbon [20]. The amount of carbon in the gas phase is indicated by the amount of carbon in the form of CH_4_ and CO_2_. Carbon in the solid phase is contained in the straw; the quantity can be calculated by subtracting the quantity in the liquid and gas phases from the initial quantity of total carbon. Based on the above assumptions, the distribution of carbon in the gas, liquid, and solid phases during AD is shown in Fig. 2a. The amount of carbon in the liquid phase rapidly increased at the beginning of AD, reaching a maximum value of 18.81 g on day 5. Thereafter, the level gradually declined until it was maintained at approximately 3 g after day 30. At the end of AD, the amount of carbon in the gas phase reached 36.78 g, whereas only 2.67 g remained in the liquid phase, indicating that most of the carbon in the liquid phase that had been hydrolyzed from the solid phase was converted into the gas phase. The amount of carbon in the solid phase changed from 76.28 to 36.83 g with a conversion rate of 51.72%, which was quite similar to the VS degradation rate of 54.57%.

Carbon exists in two forms in the gas phase, CH_4_ and CO_2_, and a floating diagram of carbon enriched in the target product CH_4_ is shown in Fig. 2b. At the beginning of AD, carbon enriched in CH_4_ was reduced by 96.55% compared with that enriched in the by-product CO_2_. As AD progressed, carbon enriched in CH_4_ continually increased. By the end of AD, on day 50, 18.46 g of carbon was enriched in CH_4_; i.e., 0.76% higher than that enriched in the by-product CO_2_.

**Fig. 3:**
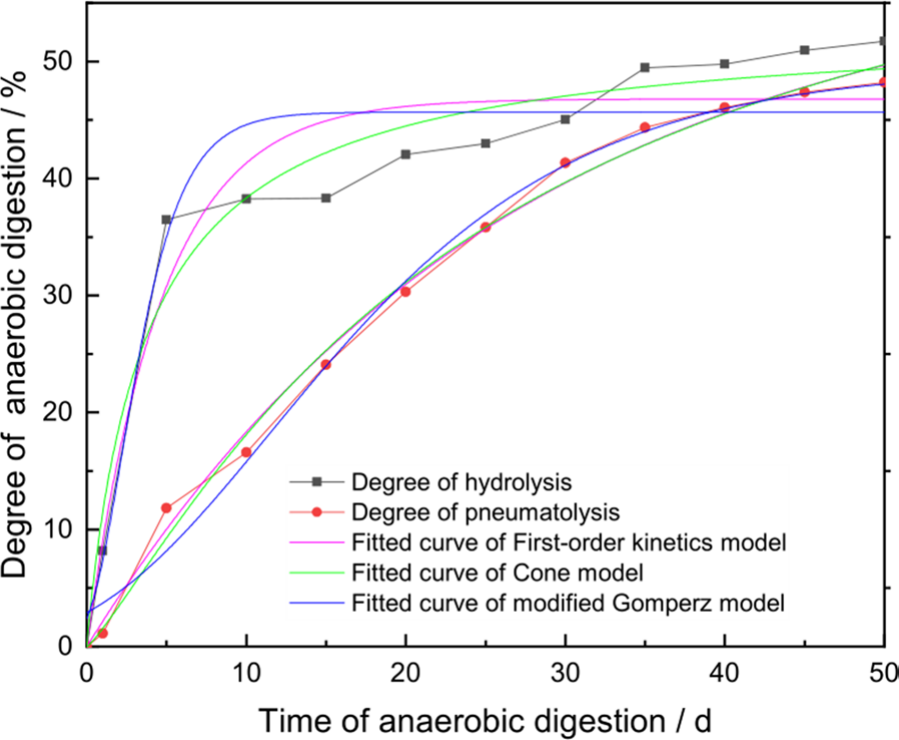
Simulation results of the degree of hydrolysis and the degree of gasification using a first-order kinetics model, a Cone model, and a modified Gompertz model

The degree of hydrolysis (DH) and degree of gasification (DG) during AD of RS along with data fitting results obtained using the first-order dynamics model, modified Gompertz model, and Cone model are shown in Fig. 3. The DH sharply increased from 8.20% to 36.49% over the first 5 days, and then the rate of increase in the DH tended to level off. At the end of AD on day 50, the DH was 51.72%. Conversely, the DG steadily increased throughout AD; however, this increasing trend slightly slowed during the final stages of AD. By the end of day 50 of AD, the DG was 48.22%. Relevant data from these dynamic models were calculated and analyzed, and the parameters estimated by the different models are shown in Table 1.

**Table 1:** Parameters estimated by a first-order kinetic model, a modified Gompertz model, and a Cone model

Degree of anaerobic digestion	Model	Parameter						Accuracy of the model	
		*P*_m_/g	*R*_m_/g	*k*/d^−1^	*λ*/d	*n*	*R*^2^	RMSPE^a^/g	AIC^b^
Degree of hydrolysis	First-order kinetic	46.8	–	0.21	–	–	0.9364	36.24	46.01
	modified Gompertz	45.67	21.99	–	0.17	–	0.9128	36.57	48.48
	Cone	53.49	–	0.26	–	0.97	0.9605	9.93	34.14
Degree of gasification	First-order kinetic	58.67		0.04	–	–	0.9947	31.83	44.58
	modified Gompertz	49.54	4.50	–	0.46	–	0.9891	35.73	48.22
	Cone	71.56	–	0.04	–	1.18	0.9934	4.35	25.07

^a^Root mean square prediction error

^b^Akaike information criterion

According to the data fitting results generated by each model, the maximum predicted DH for the AD of RS was in the range of 45.67% to 53.49%, and the Cone model predictions were the closest to the actual situation. The Cone model generated the highest R^2^ value (0.960) and exhibited a good correlation with the experimental data. Moreover, the Cone model exhibited the lowest values of root mean square prediction error (RMSPE) and the Akaike information criterion (AIC), demonstrating that it was the best of the three models for predicting the DH associated with the AD of RS.

The maximum predicted DG from the AD of RS was in the range of 49.54% to 71.56%. The modified Gompertz model output was the closest to the actual situation; the predicted maximum DG in the other two models was higher than the predicted maximum DH in the Cone model, which is not consistent with the actual condition in AD. The *R*^2^ value of the modified Gompertz model was 0.9891, demonstrating that it was the best model for predicting the DG of the AD of RS.

### Metagenomic analysis

A total of 48.81 GB of read data were generated, including 447985312 original sequences and 67645782112 original basic groups. After sequence quality control, 443321486 sequences and 66703997126 basic groups remained. High-quality sequences and basic groups comprised > 98% of the total. A total of 7484761 gene sequences were predicted, and 3447695 non-redundant gene catalogs were constructed. In specific, 533761 KEGG genes and 2684 genera were annotated.

**Fig. 4:**
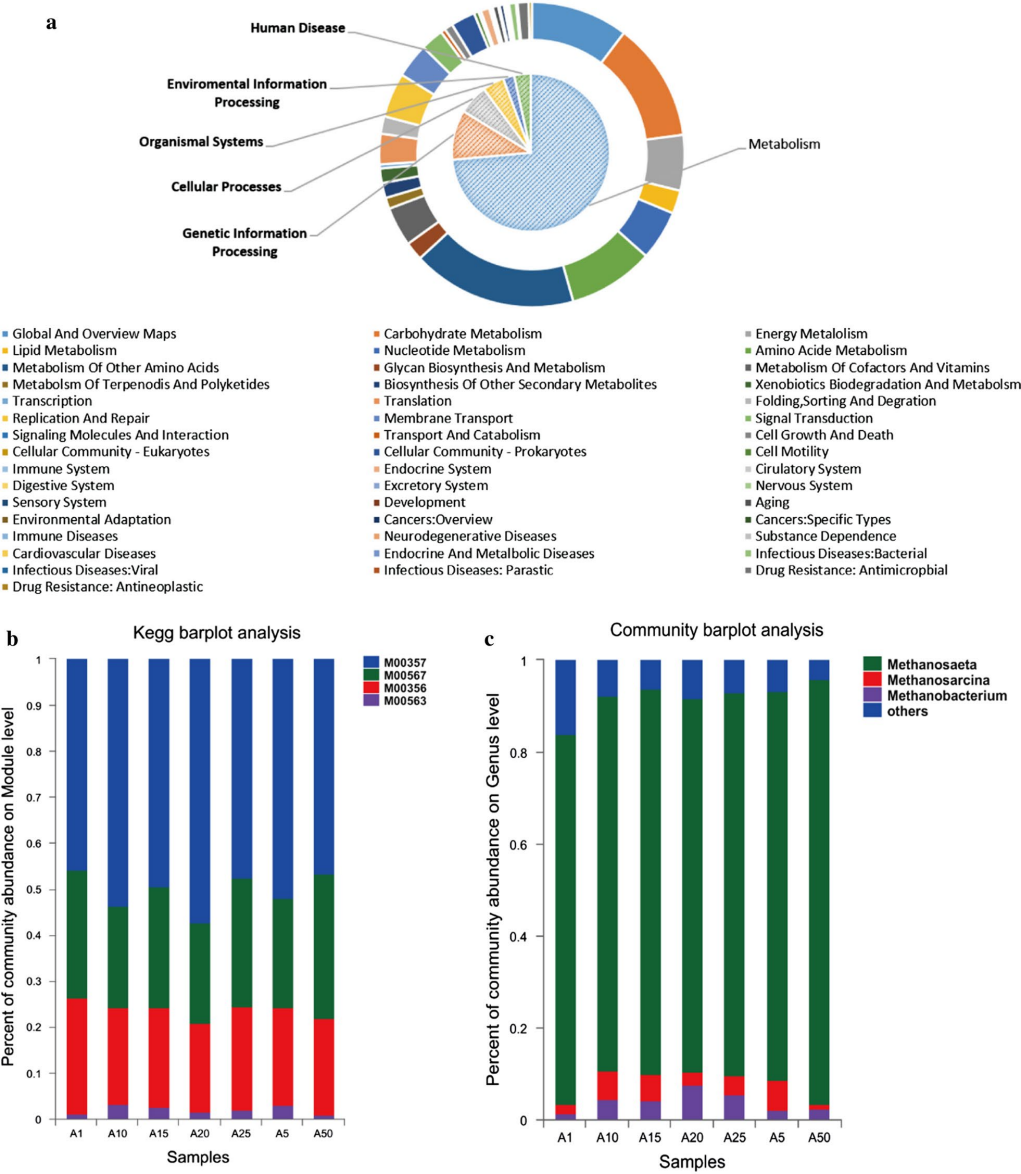
Composition of the pathways on level 1 and level 2 (**a**), modules (**b**), and archaea (c)

The composition of the pathways on levels 1 and 2 is shown in Fig. 4a. Metabolism was the dominant pathway on level 1, and Amino Acid Metabolism was the dominant pathway on level 2, which is consistent with the results reported by Li et al. [21]. Carbohydrate Metabolism was the second most dominant pathway on level 2, which is consistent with the chemical components of straw.

The pathway with the highest abundance in Carbohydrate Metabolism was Glycolysis/Gluconeogenesis (ko00010, 1.71%–1.99%), which was consistent with the conclusions obtained by Soares et al. [22]. Moreover, ko00620, ko00520, ko00630, and ko00020 were pathways with high abundance on level 3. This finding indicates that Carbohydrate Metabolism is a complex process that involves a series of complicated steps that require catalysis by multiple enzymes and is accompanied by the generation of ATP and NADH [23].

At the Module level, four Modules are related to the generation of methane: M00357, M00567, M00356, and M00563. M00357 uses acetate as a substrate. M00567 uses H_2_ or CO_2_ as a substrate. M00356 uses methanol as a substrate. M00563 uses methylamine, dimethylamine, and trimethylamine as substrates. The abundance of these four modules is shown in Fig. 4b. M00357 accounted for the highest proportion with abundance values in the range of 51.23 to 65.43% followed by M00567 with an abundance of 16.96 to 26.88%, M00356 with an abundance of 16.44 to 22.98%, and M00563 with the lowest abundance of 0.83 to 2.40%. This finding indicated that the methane production process in the present study mainly involved aceticlastic methanogenesis. The abundance of archaea at the genus level is shown in Fig. 4c. *Methanosaeta* was the most abundant archaeal genus, which uses acetic acid as a substrate [24], followed by *Methanosarcina* and *Methanobacterium*. These findings were consistent with the results of the KEGG functional analysis and further indicated that the methanogenic process in the present study mainly involved aceticlastic methanogenesis. These results are consistent with the results reported by Chen et al. [25].

**Fig. 5:**
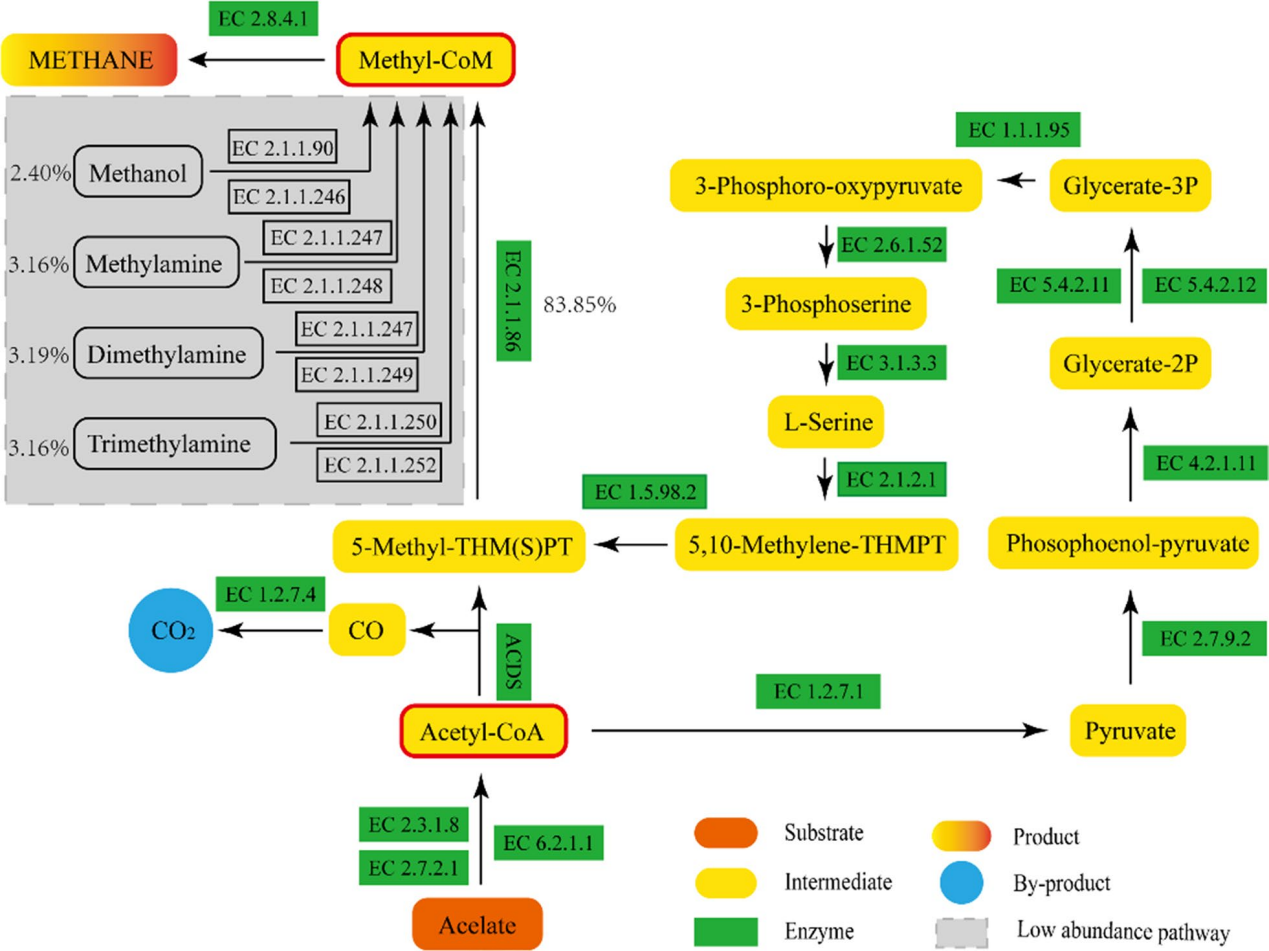
KOs with high abundance in ko00680

At the KEGG Orthologs (KO) level, 9315 KOs were obtained overall, including 141 KOs in ko00680 (Methane Metabolism). The high abundance of KOs in ko00680 is shown in Fig. 5. Methyl-CoM (C03920) is an important intermediate in the methanogenesis phase; it can be generated by a total of five biological reaction processes with a highest relative abundance of 83.85%. The biological reaction process with dominant abundance was the generation of Acetyl-CoA (C00024) from acetic acid via two biological reaction processes involving EC 6.2.1.1 or EC 2.3.1.8 and EC 2.7.2.1. Subsequently, Acetyl-CoA is converted to 5-methyl-5,6,7,8-tetrahydromethanopterin via two biological reaction processes, one of which involves a single reaction (R09096) and the other involves a series of reactions (R01196–R00199–R00658–R01513–R04173–R00582–R09099–R04464). Thereafter, Methyl-CoM is generated from 5-methyl-5,6,7,8-tetrahydromethanopterin via a biological reaction process with dominant abundance involving reaction R04347 with EC 2.1.1.86. The reaction equations are shown in Table 2.

**Table 2:** Biochemical reactions corresponding to the names of the reactions

Name	Reaction
R00235	ATP + acetate + CoA ⟺⟺ AMP + diphosphate + acetyl-CoA
R00351	Citrate + CoA ⟺ Acetyl-CoA + H_2_O + oxaloacetate
R00230	Acetyl-CoA + orthophosphate ⟺ CoA + acetyl phosphate
R09096	Acetyl-CoA + 5,6,7,8-tetrahydromethanopterin ⟺ CoA + 5-methyl-5,6,7,8-tetrahydromethanopterin + CO
R01196	2 Reduced ferredoxin + acetyl-CoA + CO_2_ + 2H + ⟺ 2 oxidized ferredoxin + Pyruvate + CoA
R00199	ATP + pyruvate + H_2_O ⟺ AMP + Phosphoenolpyruvate + orthophosphate
R00658	2-Phospho-d-glycerate ⟺ Phosphoenolpyruvate + H_2_O
R01518	2-Phospho-d-glycerate ⟺ 3-Phospho-d-glycerate
R01513	3-Phospho-d-glycerate + NAD + ⟺ 3-Phosphonooxypyruvate + NADH + H^+^
R04173	O-Phospho-l-serine + 2-Oxoglutarate ⟺ 3-Phosphonooxypyruvate + l-Glutamate
R00582	O-Phospho-l-serine + H_2_O ⟺ l-Serine + orthophosphate
R09099	l-Serine + 5,6,7,8-Tetrahydromethanopterin ⟺ 5,10-Methylenetetrahydromethanopterin + Glycine + H_2_O
R04464	5,10-Methylenetetrahydromethanopterin + reduced coenzyme F_420_ ⟺ 5-methyl-5,6,7,8-tetrahydromethanopterin + coenzyme F_420_
R04347	5-Methyl-5,6,7,8-tetrahydromethanopterin + coenzyme M + 2 sodium cation(in) ⟺ 5,6,7,8-tetrahydromethanopterin + 2-(Methylthio)ethanesulfonate + 2 sodium cations (out)

The abundance of Methyl-CoM slightly increased between days 1 and 5, and fluctuations subsequently occurred. The minimum value was reached on day 20 before Methyl-CoM began to continuously increase. This trend is strongly correlated with the trend of biogas production, except for the increase in the abundance of Methyl-CoM and the decrease in biogas production after 25 days. This finding may be due to the consumption of the substrate despite the increase in the capacity for methanation in the AD system. On the other hand, the stage at which the abundance of Methyl-CoM steadily increased was the stage at which the methane content in the biogas was becoming relatively stable. Therefore, the abundance of Methyl-CoM exhibited a strong correlation with biogas production and methane content in the AD system and may be a useful indicator to determine whether the AD system has entered the stage of stable methane production.

CO was produced during reaction R09096 with the generation of 5-methyl-5,6,7,8-tetrahydromethanopterin. CO can be converted to CO_2_ via a reaction involving EC 1.2.7.4, which is highly abundant in the AD of RS, and the relative abundances of EC 1.2.7.4 and EC 2.1.1.86, which are key enzymes of methanation, were similar to the specific values of the cumulative CO_2_ yield and cumulative CH_4_ yield. This finding is suggested that EC 1.2.7.4 played a key role in the AD of RS, generating CO_2_. However, enzymes that can use CO_2_, such as EC 1.2.7.12, EC1.17.1.9, EC 1.17.983, and EC 1.17.1.10, were present at a low abundance; thus, a limited amount of CO_2_ enters the hydrotropic methanogenic pathway, resulting in high CO_2_ content in biogas and low efficiency of methane production.

In addition to acetic acid, small molecule organic compounds (SMOC), such as propionic acid and butyric acid, are also important intermediate products in the hydrolysis and acidification stages of AD. The level at which these compounds are present can directly affect AD efficiency. At the enzyme level, 31, 7, and 5 biological processes associated with acetic acid, propionic acid, and butyric acid metabolism, respectively, were identified in the present study, and their abundances are shown in Table 3. The highest abundance was observed for enzymes associated with acetic acid metabolism followed by enzymes associated with propionic acid metabolism. A total of 20 enzymes associated with acetic acid production were identified. The total abundance of these enzymes increased significantly between days 1 and 5, decreased slightly between days 5 and 15, and then increased again until it reached the maximum value on day 25 before decreasing to the minimum value by day 50. The first 15 days showed a trend of an initial increase followed by a decrease, indicating that the production of acetic acid continued for 1–5 days. Subsequently, the microorganisms present could not consume all the acetic acid due to its increasing accumulation, resulting in the inhibition of acetic acid production and a decrease in the total amount of acetic acid produced. During the middle stages of AD, the concentration of acetic acid-generating enzymes increased, and the accumulated acetic acid was utilized by microorganisms, leading to the more stable production of methane.

**Table 3:** Enzyme abundance associated with the formation or degradation of acetic acid, propionic acid and butyrate

	Enzyme	A1	A5	A10	A15	A20	A25	A50
Acetic acid	31	2251.83	2475.83	2278.51	2450.26	2429.41	2489.79	2615.49
Acetic acid as substrate	11	808.16	837.49	854.72	951.58	1020.45	661.37	1230.52
Acetic acid as product	20	1443.67	1638.34	1423.79	1498.68	1408.97	1828.42	1384.97
Propionic acid	7	1334.17	1215.60	1343.20	1146.57	1324.50	1312.35	1527.32
Butyrate	5	1080.15	780.09	698.41	751.48	735.21	917.98	558.77

### Three stages of batch anaerobic digestion

**Fig. 6:**
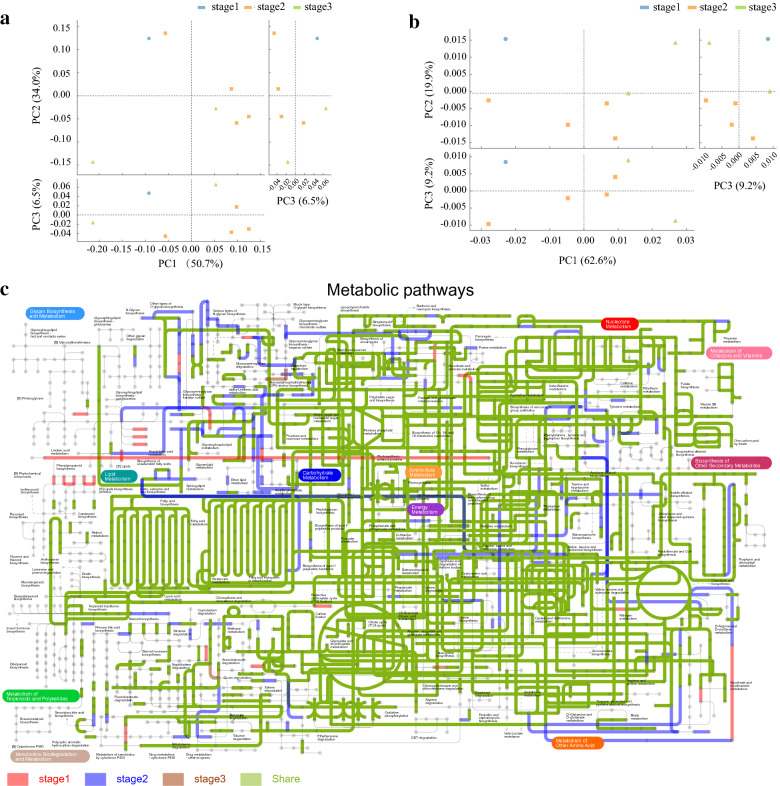
Principal component analysis (PCA) of the microbial community (**a**), PCA of the KEGG functions (**b**), and ipath analysis (**c**)

According to the biogas production performance, carbon migration, and metagenomic characteristics during the AD of RS, certain rules related to the changes in carbon content, the microbial community, and functions of this community during batch AD were observed. The beta-diversity and functions of the microbial community during the different phases of the AD of RS were analyzed. Principal component analysis (PCA) of the microbial community (Fig. 6a) showed that components in principle component axis (PC 1) can explain 50.7% of the difference of the microbial community. Components in PC 2 and PC 3 can explain 40.5% of the difference of the microbial community, illustrating that the Microbiota similarity was low. This finding suggested significant differences in the microbial community structure at the different stages of the AD of RS. Furthermore, the PCA of KEGG functions (Fig. 6b) revealed significant differences in the metabolic pathways at different stages of digestion, indicating that the pathways changed following succession in the microbial community, enabling adaptation to changes in the substrate and the digestive environment at different stages. The microbial community and functional composition of A5, A10, A15, and A20 demonstrated similar characteristics, and A25 and A50 were similar but clearly different from A0. Therefore, the entire AD could be divided into three stages: the initiation stage (days 1–5), adaptation stage (days 6–20), and stabilization stage (days 21–50).

The result of the iPath pathway analysis of the three stages is shown in Fig. 6c. Most pathways related to Amino Acid Metabolism, Energy Metabolism, Carbohydrate Metabolism, and Nucleotide Metabolism, such as Methane Metabolism, Citrate Cycle (TCA cycle), and Fatty Acid Metabolism, were active in the entire AD of RS. However, Glycan Biosynthesis and Metabolism, Lipid Metabolism, Terpenoid and Polyketide Metabolism, Xenobiotic Biodegradation and Metabolism, Cofactor and Vitamin Metabolism, Biosynthesis of other secondary metabolites and Metabolism of other amino acids, such as Glycosphingolipid Biosynthesis, Flavonoid Biosynthesis, Diterpenoid Biosynthesis, Caffeine Metabolism, and Isoquinoline Alkaloid Biosynthesis, did not occur throughout the entire AD of RS. This finding could be attributed to the lack of relevant substrates or the fact that these processes are less involved in the AD of RS.

According to the changing trends in the three stages, the unique pathways in the initiation stage included 21 pathways, such as ko00130, ko00360, ko00940, ko0059, and ko00330; the microorganisms corresponding to these pathways were inhibited or eliminated during AD and eventually disappeared. Numerous new pathways, including ko00600, ko100, ko00561, ko00564, and ko00565, appeared during the adaptation stage; however, these pathways were not adapted to an AD system and were eventually eliminated. No unique pathways were identified in the stabilization stage, indicating that the AD system had reached a stable state by this stage and that it is reasonable to divide the AD into three stages.

## Conclusions

From the perspective of carbon migration, carbon in RS was consumed (76.28–36.83 g) with a conversion rate of 51.72%. In the first 5 days, DH rapidly increased, and a large amount of carbon accumulated in the liquid phase and subsequently was converted into gas. By the end of the AD of RS, the amount of carbon in the liquid and gas phases was 2.67 and 36.78 g, respectively. From the perspective of metagenomic characteristics, the Methyl-CoM, 5-methyl-5,6,7,8-tetrahydromethanopterin and Acetyl-CoA were important intermediates during the AD of RS. According to the changes of carbon migration and metagenomic characteristics, the batch AD of RS could be divided into three stages (initiation, adaptation, and stabilization).

## Methods

Mesophilic AD of RS was performed, and the carbon migration and changes in metagenomic characteristics during the process were investigated to study the mechanisms involved in the AD of RS.

### Feedstock and inoculum

The RS was collected from Ji County (Tianjin, China). After natural air-drying, the RS was cut into 3- to 4-cm-long sections using a straw chopper. These sections were then smashed to the size of 20-mesh using a pulverizer (YSW-180). The inoculum was obtained from a biogas station in Shunyi district (Beijing). The characteristics of the raw materials and the inoculum are shown in Table 4.

**Table 4:** Specific characteristics of the raw material (rice straw) and the inoculum

Index	Rice straw	Inoculum
Total solids (TS)^a^ (%)	93.70	11.59
Volatile solids (VS)^a^ (%)	80.82	7.61
Total carbon (TC)^b^ (%)	38.14	35.15
Total nitrogen (TN)^b^ (%)	0.51	2.56
C/N	74.78	13.75
Cellulose^b^ (%)	40.44	ND
Hemicellulose^b^ (%)	29.27	ND
Lignin^b^ (%)	5.17	ND

^a^Content of fresh matter

^b^Content of dry matter

### Experimental equipment

Glass bottles with a volume of 5 L were connected to a gas-collecting device by latex and glass tubes and used as batch reactors. The discharge interface was connected by a latex tube and sealed with a water stopper.

### Experimental methods

Raw materials and the inoculum were added to the reactor; the organic loading rate of the pretreated RS was 50 gTS/L, and that of the inoculum was 20 gTS/L. Pure water was added to adjust the volume to the working volume of the AD reactor (4 L), and the initial pH in the reactor was adjusted to a value between 6.8 and 7.2 by adding Ca(OH)_2_. The AD reactor device was placed in a constant-temperature water bath at 35 °C ± 1 °C to allow mesophilic AD. The AD process was allowed to continue for 50 days. Three parallel experiments were established for each group of experiments.

### Analytical methods

Daily biogas production was measured using the water displacement method [26]. The biogas composition was determined using gas chromatography (SP-2100, BeiFenRuiLi, Beijing, China).

The American Public Health Association standard method was used to determine total solids (TS) and volatile solids (VS) [26]. The total organic carbon (TOC) content was measured by the multiple dilution method using a corrected MultiN/C3100TOC/TN; the NPOC purging time and the maximum integration time were set to 180 s.

### Degree of hydrolysis and degree of gasification

The specific quantity of carbon in the cumulative gas produced together with the amount of carbon in the liquid of the AD system and the total quantity of carbon in the RS was defined as the DH of the straw during the AD process. The specific quantity of carbon in the cumulative gas produced together with the total quantity of carbon in the RS was defined as the DG of the straw during the AD process. A brief description of DH and DG is shown in Eqs. 1 and 2:

1 $${\text{DH}}(\% ) = \frac{C(g) + C(l)}{C(t)} \times 100\%$$

2 $${\text{DG}}\left( \% \right) = \frac{C(g)}{C(t)} \times 100\%$$

where *C*(g) is the amount of carbon in the cumulative gas produced (unit: g); *C*(l) the amount of carbon in the liquid phase (unit: g); and *C*(*t*) the amount of the carbon in the RS material (unit: g).

### Dynamic analysis

A first-order reaction kinetics model [27], the modified Gompertz model [28], and the Cone model [29] are the most widely used models for the analysis of AD of complex organic matter. These models are typically used to analyze the degree and rate of organic matter degradation. DH and DG were fitted by the three models.

The formula of the first-order dynamic model is shown in Eq. 3:

3 $$P_{(t)} - P_{\text{m}} \left( {1 - \exp \left( { - kt} \right)} \right)$$

where *P*_(*t*)_ is the degree of hydrolysis or of gasification of the material on day *t* (%); *P*_m_ the maximum degree of hydrolysis/gasification (%); *k* the first-order reaction constant (day^−1^); and *t* the AD time (days).

The modified Gompertz model formula is shown in Eq. 4:

4 $$P_{(t)} = P_{\text{m}} \times \exp \left\{ {1 - \exp \left[ {\frac{{R_{\text{m}} }}{{P_{\text{m}} }}} \right]\left( {\lambda - t} \right) + 1} \right\}$$

where *P*_(*t*)_ is the degree of hydrolysis/gasification of the material on day t (%); *t* is the digestion time (d); *P*_m_ is the maximum degree of hydrolysis/gasification (%); *R*_m_ represents the maximum hydrolysis/gasification rate (%); *λ* is the delay time (days); and e is the natural constant, 2.71828.

The Cone model formula is shown in Eq. 5:

5 $$P_{\left( t \right)} = \frac{{P_{\text{m}} }}{{1 + \left( {kt} \right)^{ - n} }}$$

where *P*_(*t*)_ is the degree of hydrolysis/gasification of the material on day t (%); *P*_m_ is the maximum degree of hydrolysis/gasification (%); *k* represents the rate constant (d^−1^); *n* is the shape factor (dimensionless); and *t* is the digestion time (days).

The applicability of the models was evaluated by the root mean square percentage error (RMSPE) and the Akaike information criterion (AIC) [30].

The RMSPE was calculated using Eq. 6:

6 $${\text{RMSPE}} = \sqrt {\sum\nolimits_{i = 1}^{n} {\frac{{\left( {Pv_{i} = Mv_{i} } \right)^{2} }}{n}} }$$

where *Pv*_*i*_ represents the degree of hydrolysis/gasification (%); *Mv*_*i*_ represents the measured degree of hydrolysis/gasification (%); and n is the number of measurements.

AIC was calculated using Eq. 7 and was used to represent the degree of information missing from the model:

7 $$AIC = n\ln \left( {\frac{\text{RSS}}{n}} \right) + 2\left( {N + 1} \right) + \frac{{2\left( {N + 1} \right)\left( {N + 2} \right)}}{{\left( {n - N - 2} \right)}}$$

where RSS represents the sum of the squares of the residuals; *n* is the number of data points; and *N* is the number of model parameters.

### Metagenomic analysis method

Seven samples collected on inoculum and on the 5th, 10th, 15th, 20th, 25th, and 50th days during the AD of RS; these samples were designated A0, A5, A10, A15, A20, A25, and A50, respectively.

DNA was extracted using a FastDNA^®^ SPIN Kit for Soil (Omega Bio-tek, USA). After the genomic DNA had been extracted, its concentration was determined using TBS-380, its purity was determined using a NanoDrop 2000, and its integrity was determined using 1% agarose gel electrophoresis. DNA was segmented by Covaris M220 (gene company, China), and fragments approximately 400 bp in length were screened. A PE library was constructed using a NEXTFLEX Rapid DNA-Seq (Bio Scientific, USA) library building kit. Following PCR amplification, the macrogenome was sequenced using the Illumina novaseq/hiseq X Ten (Illumina, USA) sequencing platform.

Fastp was used to control the quality of the original data. BWA was used to compare the reads with the DNA sequence of the host, and polluted reads with high similarity were removed. MEGAHIT [31] was used to splice and assemble the optimized sequences. Contigs in the splicing results of ≥ 300 bp were selected as the final assembly result. MetaGene [7] was used to predict the open reading frame (ORF) of the assembled contig, and CD-HIT [32] was used to cluster the predicted gene sequences of all samples to build a non-redundant gene set. Finally, high-quality reads of each sample were compared with non-redundant gene sets (95% identity) using SOAAPaligner [33], and the abundance of genes in the corresponding samples was determined.

Representative sequences of the non-redundant gene catalog were aligned with the NCBI NR database [34] and the Kyoto Encyclopedia of Genes and Genomes (KEGG) database [35] for taxonomic annotation and KEGG annotation, respectively.

### Data analysis

The composition of microbial communities and their functions were analyzed using ggplot2 packages of R language (for Windows 3.5.1). PCA was performed using STAMP (Statistical Analysis of Metagenomic Profiles). The metabolic pathway data were analyzed using iPath2.0 (http://pathways.embl.de).

## Supplementary information

**Additional file 1.** Abundance of functions on level 1, level 2 and level 3.

**Additional file 2.** Abundance of functions on KO level.

**Additional file 3.** Abundance of functions on enzyme level.

## Data Availability

All data generated or analyzed during this study are included in this manuscript and its Additional files 1, 2 and 3.

## Abbreviations

AD: Anaerobic digestion
RS: Rice straw
TS: Total solids
VS: Volatile solids
TOC: Total organic carbon
VFA: Volatile fatty acid
CSTR: Continuously stirred tank reactor
TCD: Thermal conductivity detector
PCA: Principal component analysis
STAMP: Statistical analysis of metagenomic profiles
RMSPE: Root mean square prediction error
AIC: Akaike information criterion
DG: Degree of gasification
DH: Degree of hydrolysis
KEGG: Kyoto Encyclopedia of Genes and Genomes
